# Deciphering decarbonization trajectories in China by spatiotemporal-accumulation modeling of electricity carbon footprint

**DOI:** 10.1016/j.isci.2025.111963

**Published:** 2025-02-06

**Authors:** Jing Tang, Rui Shan, Peng Wang, Wei-Qiang Chen, Dungang Gu, Guanghui Li, Pinhua Rao, Jinguo Wang, Jiaqi Lu

**Affiliations:** 1 Innovation Centre for Environment and Resources, Shanghai University of Engineering Science, No.333 Longteng Road, Songjiang District, Shanghai 201620, China; 2 Department of Environmental Sciences and Engineering, Gillings School of Global Public Health, University of North Carolina at Chapel Hill, Chapel Hill, NC, USA; 3 Key Lab of Urban Environment and Health, Institute of Urban Environment, Chinese Academy of Sciences, Xiamen, Fujian 361021, China

**Keywords:** Environmental science, Energy policy, Engineering, Energy management

## Abstract

Recent climate-trade policies emphasize managing carbon embedded in goods, with electricity carbon footprints as a key metric. However, the rapid energy transition complicates this evaluation. An innovative spatiotemporal model, accounting for dynamically-installed low-carbon energy infrastructure (LCPI), was developed to assess electricity decarbonization trajectories. Applying to Chinese power grid, the national and provincial electricity carbon footprints are projected to analyze the impact of LCPI deployment. By 2050, under aggressive decarbonization scenarios, the electricity carbon footprint is projected to reach 0.12 kg CO_2_-eq/kWh—a 38.13% reduction compared to scenarios that ignore LCPI decarbonization, and a 19.18% difference when neglecting the heterogeneous carbon footprint of spatiotemporally accumulated LCPI. Meanwhile, up to 57.94% of China’s electricity carbon footprint stems from historical LCPI production emissions, thereby stressing the long-lasting impact of renewable investments. Such insights support targeted policies to systematically reduce energy-related carbon footprints, providing a scalable roadmap for global sustainable energy practices.

## Highlights

• Future product carbon footprint of Chinese grid with high spatiotemporal resolution
• Cumulation of low-carbon power infrastructure (LCPI) with various carbon footprints
• Synergistic decarbonization effect between power grid and LCPI
• Insights into systematic reduction of the carbon footprint of the power system

## Graphical abstract

**Figure:**
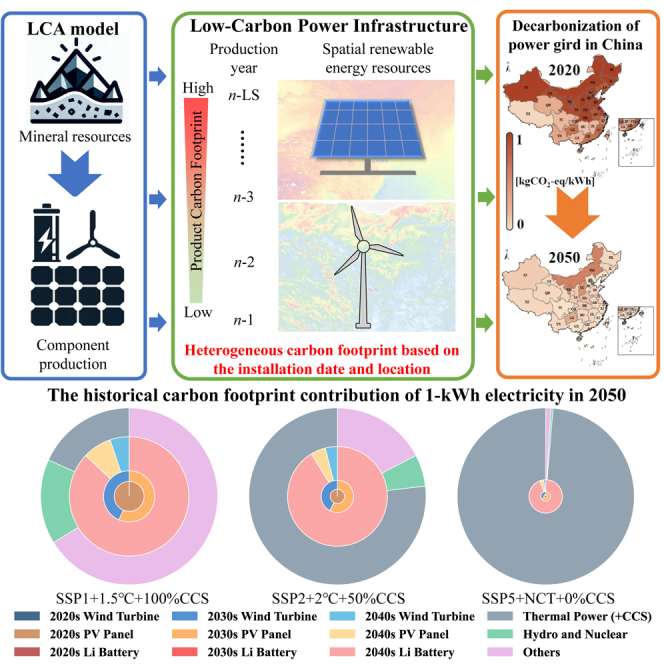

## Introduction

In the context of a global consensus aimed at combatting climate change, COP 28 emerged as a landmark event, formalizing a commitment to transition away from fossil fuels,^1^ and striving to uphold the Paris Agreement’s 1.5°C goal within reach.^2^ Key achievements during the conference include establishment of the Loss and Damage Fund, emphasis on renewable energy production, and the launch of the Climate Club. Members of the Climate Club will work together to address climate challenges and strengthen the link between climate action and international trade by creating a unified carbon market and promoting more flexible trade policies.^3^ Aligning with COP developments and the implementation of more climate-related trade policies, the intersection between trade and climate is anticipated to become increasingly significant in the future.

In recent climate trade policies, the famous ones are the carbon border adjustment mechanism (CBAM) and the new EU battery regulation.^4^^,^^5^ CBAM aims to levy tariffs on imported products with high embedded greenhouse gas (GHG) emission to prevent the leakage of carbon market. The EU battery regulation stipulates that only batteries with established life cycle GHG footprint declarations can be placed on the market. These initiatives signify a paradigm shift from organizational carbon management to a product life cycle approach for GHG emissions management, emphasizing the critical importance of assessing the carbon footprint of electricity and related infrastructure, given their central role in nearly all manufacturing processes.

China has undertaken a rapid shift toward a renewable energy-based grid,^6^^,^^7^^,^^8^ along with manufacturing considerable amount of low carbon power infrastructure (LCPI) including solar photovoltaic (PV) panels, wind turbines, and lithium battery storage. Projections from Chinese government affiliated research institute indicate that by 2050 wind power generation will reach 53,500 billion kilowatt-hours and solar power generation will reach 43,100 billion kilowatt-hours.^9^ However, from a life cycle perspective, the expansive growth of LCPI will inevitably contribute to a substantial amount of GHG emissions, resulting from the manufacturing process.^10^^,^^11^ At the same time, the decreasing carbon footprint of electricity due to the operation of LCPI, can further reduce the carbon footprint calculations of LCPI^12^ and almost all products,^13^ complicating the carbon footprint calculation. The dramatic transformation in China’s grid and extensive global trading activities make it a compelling case for analyzing the dynamics of energy system carbon footprints in the context of climate trade policy.

The carbon footprint of China’s electricity system and the future evolution of China’s power system has long been two subjects of great interests but research at their future environmental footprint is rare. As for the carbon footprint, Chen et al. have built a topological network to calculate the life cycle GHG emissions, of the electricity supply system in China in 2010.^14^ Later, researchers have analyzed electricity carbon footprint from specific technology. For example, Ruan et al. computed the electricity carbon footprint from wind power in China.^15^ Gao et al. calculated carbon footprint of coal-fired power plants from 2000 to 2020 in China.^16^ Future evolution of China’s power system and related emission have received enduring attention. For instance, Wang et al. comprehensively modeled how China utilize solar, wind and energy storage to achieve carbon neural in the electricity system while also assessed this impact on poverty, land and total system cost.^17^ Instead of a deterministic model, Zhang and Chen adopted a probabilistic model to simulate future electricity system in China.^18^ However, for a long time, there is limited attempt to analyze the future electricity carbon footprint in China considering the rapid evolution of energy transition pathways.

The shift in GHG emission management necessitates the adaptation of related modeling tools, such as a more accurate carbon footprint modeling tool, to guide climate policy and to reflect the decarbonization efforts. The current methods of calculating carbon footprint of electricity are not capable to reflect a rapid GHG emission reduction occurring. For example, existing literature often considers the carbon footprint of renewable energy generation on a national or annual basis,^19^^,^^20^^,^^21^^,^^22^^,^^23^^,^^24^ rather than capturing the dynamic decarbonization process of a power grid over continuous years. While some dynamic models exist, such as premise tool and the one by Emmott et al.^25^ for photovoltaic technology. They often overlooked the heterogeneity of GHG emissions in the production process of photovoltaic components which updated the carbon footprint of all solar PV panels in the grid and ignored the fact that they were manufactured in different years. Timex considered the heterogeneity but it failed to capture the interaction between electricity and almost all components in the carbon footprint calculation, usually resulting in a declining trend of electricity carbon footprint during decarbonization.^26^ Consequently, there is a pressing need for a methodology that accurately reflects the carbon footprint of the power system and related products. Such a methodology would encourage all industrialized nations to further promote grid decarbonization through enhanced competitiveness of exported goods regarding the carbon footprint-oriented climate trade policies.

Future transformation in China’s energy system depends on many factors, especially the socioeconomic development, climate policy and technology advancement such as carbon capture and storage technology. To capture the uncertainty of decarbonization pathways, we conduct the same analysis under five different shared socioeconomic pathways (SSPs) as shown in Table S1 of supplemental information (SI), under three different climate targets, i.e., No climate targets (NCT), 1.5°C and 2°C, under different CCS scenarios (Table 1), and the mix of all these scenarios.

**Table 1:** Qualitative description of different CCS installation ratios in 2050

	No CCS	25%CCS	50%CCS	75%CCS	100%CCS
Description	CCS is not installed	25% of coal-fired power plants are equipped with CCS	50% of coal-fired power plants are equipped with CCS	75% of coal-fired power plants are equipped with CCS	All thermal power plants are equipped with CCS

**Figure 1:**
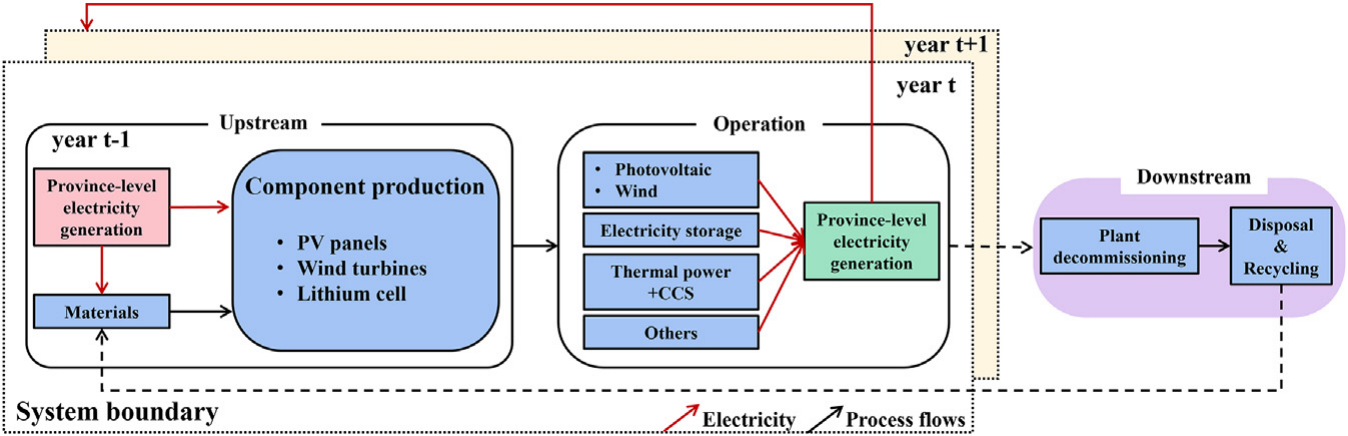
Schematic system boundary for modeling the carbon footprint of LCPI for electricity generation with spatiotemporal variations

In this paper, we extend the heterogeneous embedded carbon (HEC) dynamic life cycle assessment (LCA) model^27^ from focusing solely on the cradle-to-gate production of PV panels to a comprehensive electricity grid system. This expanded model incorporates renewable energy supply, energy storage systems, and CCS integration for thermal power plants. By considering various energy transition scenarios in China, we project the electricity product carbon footprint across different energy sources and production mixes for provincial power grids (Tables S2 and S3 in supplemental information). This advanced model captures the heterogeneous and accumulated carbon footprint of annually installed LCPI, deciphering decarbonization trajectories of the power grid toward carbon neutrality. As depicted in Figure 1, firstly, based on the life cycle inventory of LCPI in the Ecoinvent^28^ database, the electricity consumption from cradle to gate for wind turbines, photovoltaic panels, and lithium batteries is extracted. Then, two baselines and one proposed model (Table 2) are calculated and compared to illustrate the effect of LCPI decarbonization in carbon footprint calculation. Finally, based on the provincial-level electricity production structure prediction model developed by Li et al.,^29^ the carbon footprint of China’s provincial grid from 2020 to 2050 is calculated. For detailed information, please refer to STAR Methods. The final results not only provide carbon footprint of electricity, but also facilitate companies especially the outward-oriented enterprises to plan for the future compliance costs^30^^,^^31^ in dealing with carbon tariffs and other climate trade policy.

**Table 2:** Qualitatively describe three scenarios of indirect greenhouse gas emissions (GHG_ele_) from cradle-to-gate power consumption in LCPI component production

Scenario	Mathematical expression	Brief description
BS1	${GHG}_{\text{ele}}\left(p,t\right)\equiv {GHG}_{\text{ele}}\left(p,2020\right)$	Baseline reflecting the current product carbon footprint of new components, sourced from the Ecoinvent database.
BS2	${GHG}_{\text{ele}}\left(p,t\right)=E_{LCPI}\times {ECF}_{\text{nat}}\left(t-1\right)$ ${ELE}_{LCPI-new}\left(p,t\right)\equiv {ELE}_{sum}\left(p,t\right)$	The decarbonization process of electricity used in the cradle-to-gate production of LCPI components is incorporated into calculation. The calculated results are used for all LCPI components presented in the system.
HEC Model	${GHG}_{\text{ele}}\left(p,t\right)=E_{LCPI}\times {ECF}_{\text{nat}}\left(t-1\right)$ ${ELE}_{LCPI-new}\left(p,t\right)={ELE}_{sum}\left(p,t\right)-{ELE}_{sum}\left(p,t-1\right)$	The carbon footprint of LCPI components considers the decarbonization of electricity grid. Moreover, the footprint of LCPI differs by their installation year. These factors are then accounted into grid footprint calculation.

*E*_LCPI_ refers to the electricity consumed in the manufacturing process of LCPI components. *ECF*_nat_ mean the national average electricity carbon footprint. *ELE*_LCPI-new_ mean the annual power generation of newly installed LCPI. *ELE*_sum_ represent the total power generation by LCPI. The *p* and *t* represent the province and time of LCPI production.

## Results

### The synergistic impact of low carbon power infrastructure and grid decarbonization on electricity carbon footprint

**Figure 2:**
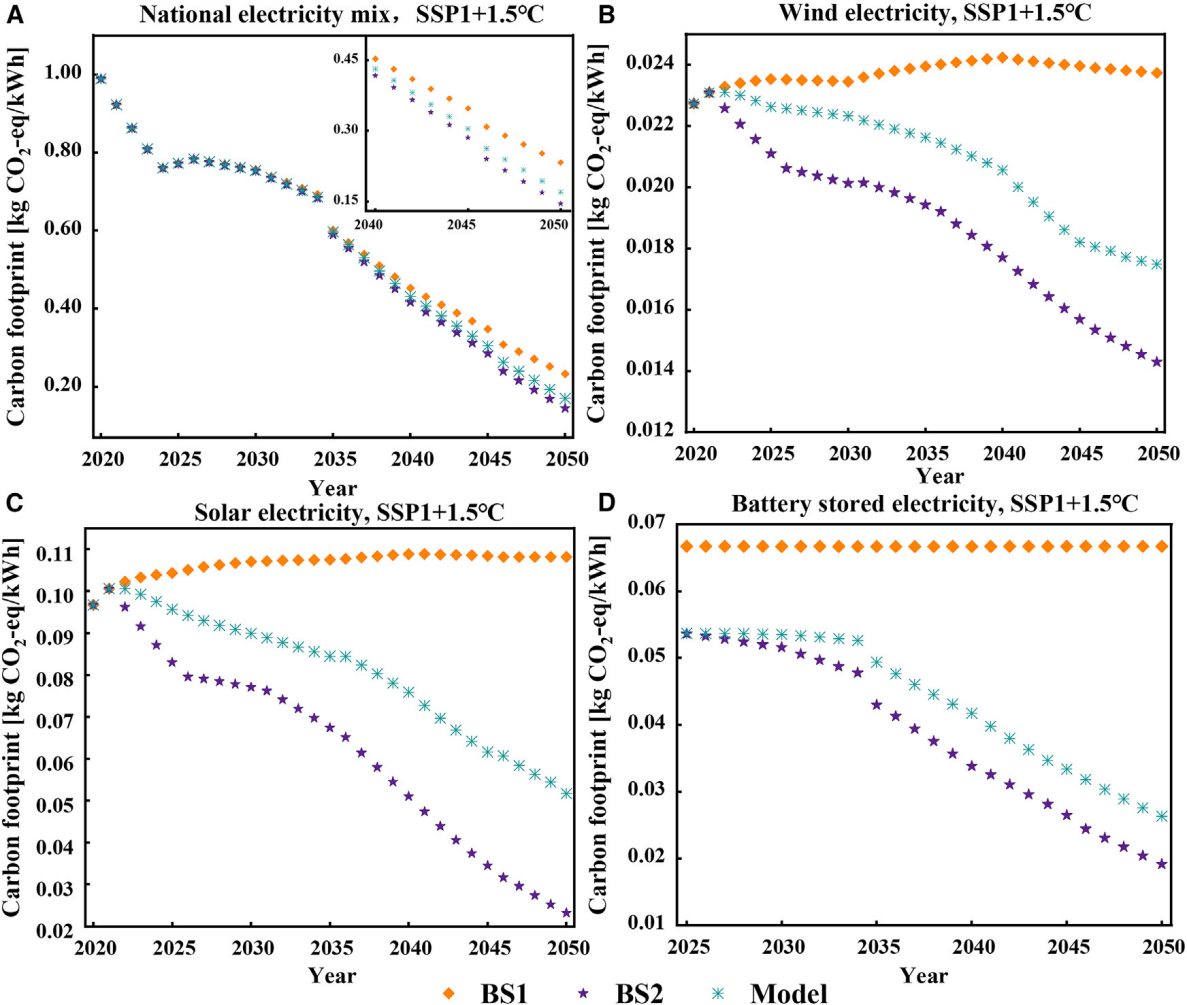
Modeled carbon footprints under various methods (A) electricity mix from total national generation portfolio; (B) national average of wind electricity; (C) national average of solar electricity; (D) national average of battery-stored electricity.

The comparative analysis presented in Figure 2 elucidates the disparities among the outcomes derived from two baseline scenarios (BS1 and BS2) and the proposed HEC model when adopting the sustainable development pathway to achieve the 1.5°C target (SSP1+1.5°C). In Figure 2A, the carbon footprints of China’s national electricity mix were compared among BS1, BS2, and the proposed model, revealing significant differences. Overall, the decarbonization trend is the same for all three scenarios: a significant reduction in the national electricity mix carbon footprint from 2020 to 2025, followed by a slowdown from 2025 to 2035 until it begins to re-accelerate in 2036, eventually reducing to less than 0.15 kg CO_2_-eq/kWh. The sudden jump at 2025 is caused by a massive deployment of battery storage which is assumed in the grid expansion model based on existing national policies (Table S4 and Figure S1 in supplemental information).

However, as the decarbonization process progresses, the differences among the three scenarios become apparent. BS1 adopts a constant carbon footprint of LCPI which did not consider the decarbonization. With the expansion of LCPI, more green electricity is injected into the grid, leading to the lower carbon footprint of LCPI. This pheromone is captured by the HEC model. Compared to the HEC model, BS1 differs by up to 26.94% (2050), a change due to the synergistic decarbonization effect between the LCPI and the power sector.

On a separate note, BS2 exhibits a notably lower electricity carbon footprint compared to both BS1 and the model, with a maximum difference of 14.53%. This reduction aligns with expectations, as BS2 calculates the manufacture carbon footprint of all LCPIs using the most recent electricity footprint, overlooking the carbon-intensive electricity used in manufacturing previous LCPIs. This study only considered the decarbonization of electricity. If the model includes more decarbonization paths, the historical cumulative effect of old LCPI with high carbon footprints will become more apparent, indicating that the BS2 overestimates the decarbonization potential of electricity. The overestimated decarbonization in BS2 underscores the importance of time-matching requirements in carbon footprint calculations for goods. It is optimal to align input elements, such as electricity, with data estimates from the closest, reasonably recent year, avoiding extremes of recency or age.

Figures 2B–2D illustrates the carbon footprint per kWh for wind, solar, and battery-stored electricity, revealing the differences among three methods of modeling electricity carbon footprints. While China’s national electricity mix shows slight variation when considering the decarbonization of the LCPI industry chain alongside the grid, the discrepancies among the three algorithms become significant when analyzing individual carbon footprint of LCPIs. Despite this, the overall changing trend toward decarbonization remains consistent with the national electricity mix. According to the proposed HEC model, by 2050, the electricity carbon footprints for these three sources will increase by 18.27%, 55.08%, and 27.32%, respectively, compared to baseline scenario BS2. As explained before, such a difference results from the assumption that all solar or wind panel are made in the same year with the same electricity carbon footprint without considering the heterogeneity. The disparities in LCPI carbon footprints among scenarios significantly outweigh disparities in electricity carbon footprints. This is due to the fact that the overall electricity supply still heavily relies on fossil fuels, which remain unchanged despite efforts to decarbonize electricity generation. Consequently, this dilutes the potential synergistic impact of decarbonization efforts on both electricity and LCPIs. When fossil fuels are reduced or even eliminated in the future, it will be crucial to pay attention to the impact of LCPI’s historical carbon accumulation on the grid’s carbon footprint.

### The decarbonization of electricity carbon footprint under various SSPs

**Figure 3:**
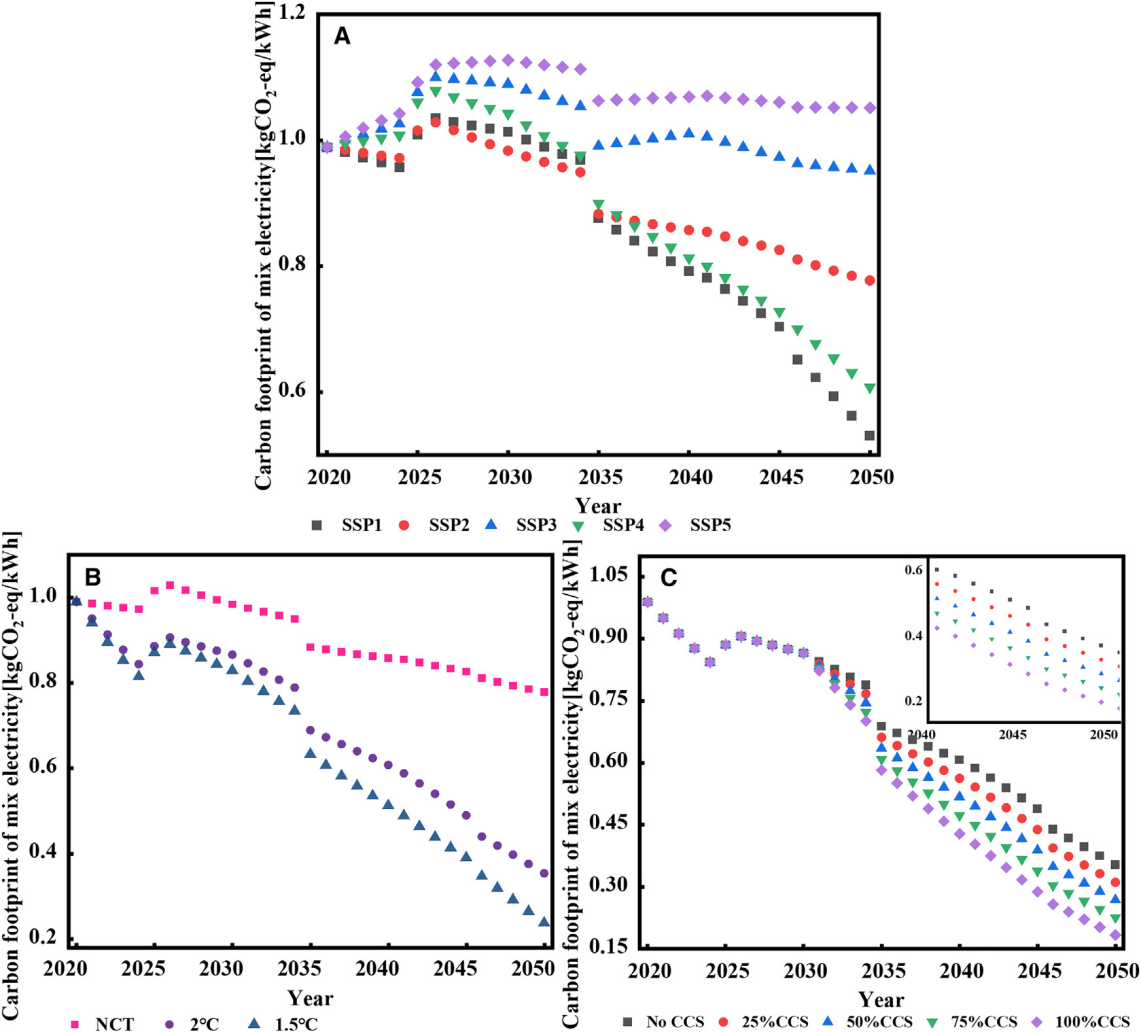
Changes in the carbon footprint of national electricity mix (A) The effect of various shared socio-economic pathway (SSPs) without climate targets; (B) The impact of different climate targets under SSP2; (C): The impact of different proportions of CCS installation (penetration rate in 2050) under the SSP2 scenario of 2°C. (see excel file Table S14 in supplemental information).

As previous results have already demonstrated the advantage of the proposed HEC model, following chapters capture the uncertainties of carbon footprints calculated by HEC model under different scenarios. Figure 3A emphasizes the considerable influence of different SSP trajectories without climate targets on the carbon footprint of China’s power sector.

Under SSP1 (sustainable pathway), lower electricity demand coupled with advanced in renewable energy technology significantly lowers the carbon footprint of electricity (*ECF*_nat_: 0.53 kg CO_2_-eq/kWh by 2050). In this scenario, renewable energy sources, such as wind, solar, and nuclear power will rapidly replace thermal power generation after 2025. The sudden leap in 2025 is caused by the large-scale deployment of battery storage assumed in the grid expansion model based on existing national policies. Additionally, every ten years, the batteries are completely replaced, leading to another small leap in 2035. As the batteries become increasingly eco-friendly, the fluctuations in the carbon footprint of the grid also gradually decrease.

SSP2 (middle of the road) presents a gradual increase in LCPI, thereby slowly diminishing reliance on coal-fired power, resulting in a carbon footprint of 0.78 kg CO_2_-eq/kWh in 2050. Contrary to popular expectations,^32^ due to the cumulative effects of LCPI, the swift deployment of renewable energy in SSP1 within a short period of time initially leads to a higher carbon footprint compared to SSP2, which persists until 2035. In other words, in SSP1 scenario, it will take ten years for the renewable energy generation system to deliver GHG emission reduction benefits to the power gird from a carbon footprint perspective, taking SSP2 as the benchmark.

Under the SSP3 (regional rivalry), escalating regional conflicts and a trend toward deglobalization have increased the difficulty of reducing GHG emissions and adapting to climate change goals.^33^ Meanwhile, the growth in electricity demand in China, which is three times higher in 2050 compared to 2015,^29^ poses challenges to the development of low-carbon power generation facilities. As a result, there is a heavy reliance on fossil fuels to meet energy demands, leading to a consistently high carbon footprint of around 1.02 kg CO_2_-eq/kWh.

SSP4 (Inequality pathway) demonstrates considerable development disparities globally, and the carbon footprint of electricity will decrease to 0.61 kg CO_2_-eq/kWh by 2050. SSP5 (fossil-fueled pathway) as a scenario of low adaptation and high mitigation, has a high dependence on traditional fossil fuels and a high electricity demand, making it the development pathway with the highest carbon footprint, consistently around 1.07 kg CO_2_-eq/kWh.

Compared to conventional studies that focus on the trajectories of macro-level GHG emissions associated with SSP variations, our research adopts a more systematic approach from the perspective of carbon footprint to evaluate grid decarbonization. It not only emphasizes the energy structure of power grid—an aspect underscored in carbon intensity discussions—and enhances this framework with the inclusion of the decarbonization effects of LCPI, thereby addressing the “Scope 3” emissions impact.

### The influence of climate targets and technology advance

Then, the study looks into the political environment as represented by different climate targets which are NCT, 1.5°C, and 2°C target. As shown in Figure 3B, taking the intermediate pathway (SSP2) as an example, under the three climate targets, renewable energy will account for 45.68%, 86.71%, and 96.61% of the total electricity in 2050, respectively. Therefore, under the 2°C and 1.5°C scenarios, the carbon footprint of electricity can be reduced to 0.35 and 0.24 kg CO_2_ eq/kWh, respectively, which is 54.54% and 69.56% lower than that of NCT, respectively.

China, with strong political will in sustainable development, also proposed its climate target^34^ that the whole society aims to achieve carbon neutrality by 2060. To achieve the 2060 target, the State Grid Corporation of China aim to deeply decarbonize the grid by 2050.^35^ This 2050 target in Chinese electricity system align with the global 1.5°C target according to previous analysis.^36^ Since this study only analyze the electricity system rather than the whole society, we can regard the Chinese 2060 target the same as the 1.5°C target in this study.

This comparative analysis underscores that the importance of policy target. It necessitates the strategic implementation of policy measures, as the reliance on technological cost benefits alone (via market mechanisms) is insufficient. Consequently, the formulation of explicit policy targets is imperative for the realization of these ambitious climate goals.

Although CCS is currently costly and technically uncertain, it is regarded as an effective way to reduce emissions from thermal power generation, especially to deeply decarbonize (<0.20 kg CO_2_-eq/kWh) the power sector. Utilizing the life cycle dataset of CCS operation phase released by Volkart,^37^ we calculated the carbon footprint of thermal and biomass power plants integrated with CCS in China. Based on the literature review,^38^ it is assumed that large-scale deployment of CCS will begin in 2030, increasing linearly to 25%, 50%, 75%, and 100% by 2050. This allows us to explore in depth the impact of technological change on the carbon footprint of the national electricity mix. As demonstrated in Figure 3C, under the SSP2 path guided by the 2°C temperature target policy, the power sector can achieve deep decarbonization by adopting a high proportion of CCS technologies. The installation of CCS technologies at increasing proportions leads to a sequential reduction in the carbon footprint of electricity by 12.01%, 13.65%, 15.80%, and 18.77% in 2050, compared to the scenario without CCS installation. Specifically, when the installation proportion of CCS reaches 75%, the carbon footprint of electricity is reduced to 0.23 kg CO_2_-eq/kWh in 2050, which is comparable to the levels targeted in the 1.5°C scenario. This indicates that the widespread application of CCS can effectively decarbonize the power grid, achieving similar outcomes to those expected under stringent climate policies. This also highlights the synergistic effect of combining carbon reduction technologies with climate policies.

### The divergence among calculation models under different scenarios

**Figure 4:**
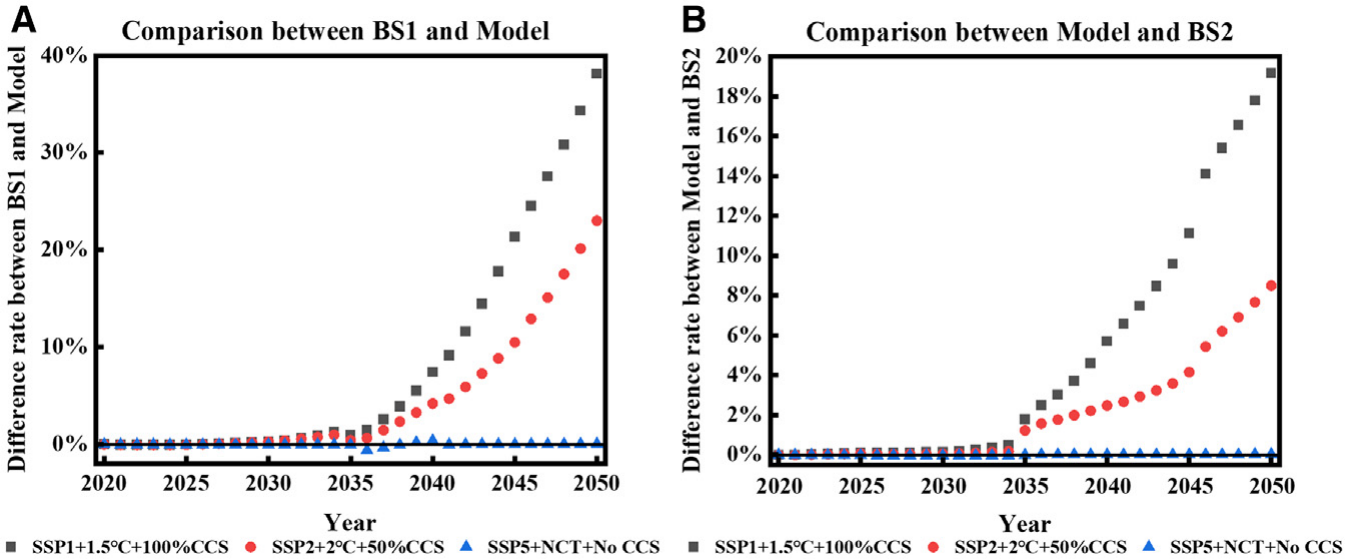
The difference rate between the model and the two baseline scenarios under different scenarios (A) Compared the differences between BS1 and Model; (B) Compared the differences between Model and BS2.

Three pathways were selected to further explore the divergences among different calculation models for the electricity carbon footprint. They are the low-carbon (SSP1+1.5°C+100%CCS), high-carbon (SSP5+NCT+No CCS) scenarios, and the medium scenario (SSP2+2°C+50%CCS). Shown in Figure 4, the more active in decarbonization, the greater the difference between the algorithms is. Compared with BS1, the HEC model always has a lower carbon footprint in electricity. This difference and its enlarging trend can incentivize the decarbonization due to the fact that the HEC model can better capture the efforts in decarbonization. The legacy issues of LCPI production from previous years have consistently resulted in the HEC model’s electricity carbon footprint being higher than that of the BS2. Meanwhile, the acceleration of the pace of decarbonization magnifies the difference between HEC and BS2, with a maximum difference of 19.18% under the low-carbon scenario, which further highlights the importance of accounting for historical emissions responsibilities.

#### The contribution analysis of electricity carbon footprint under various scenarios

**Figure 5:**
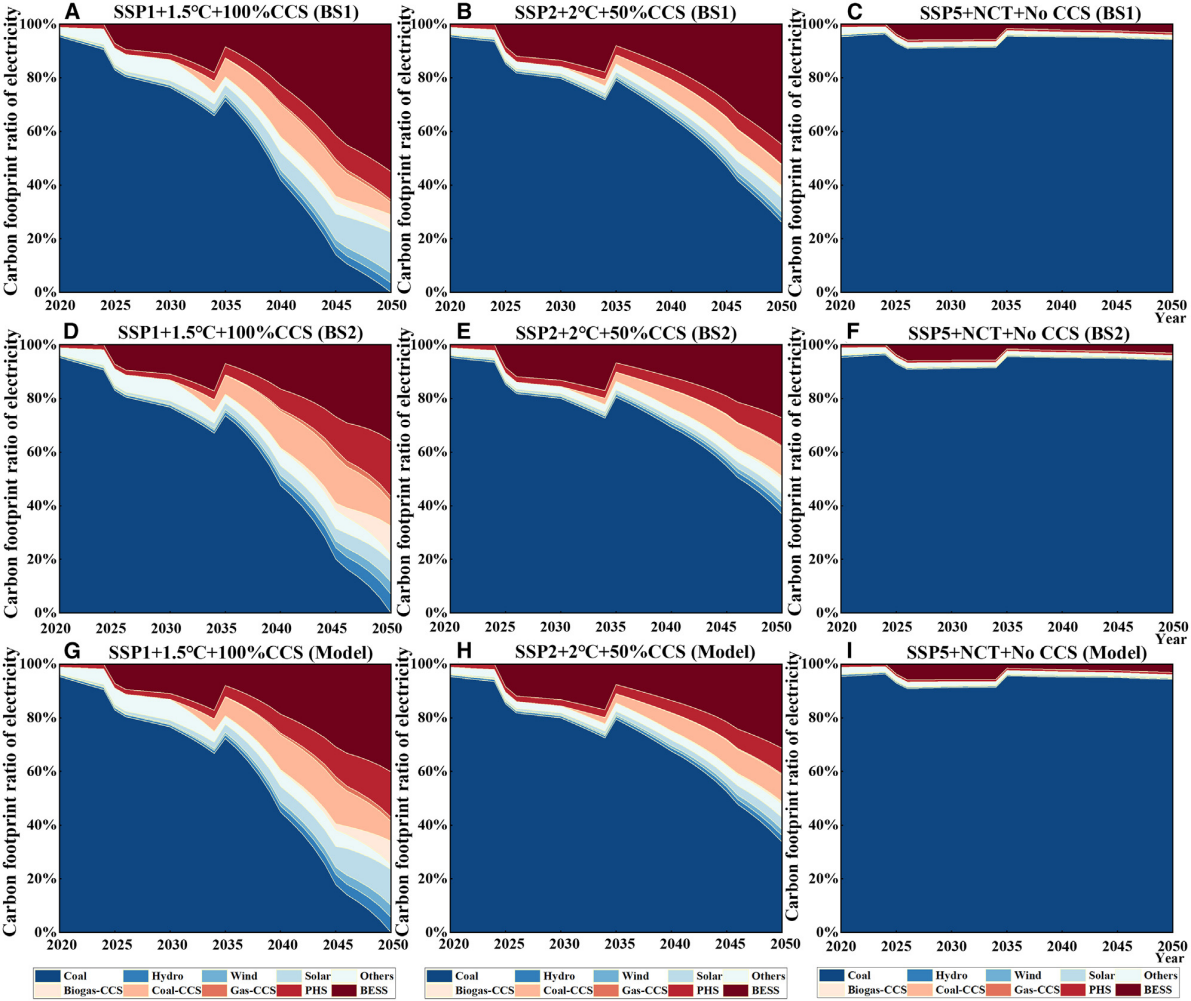
Changes in the contribution of different energy sources to the carbon footprint of national electricity mix (A–C) The impact of the three integrated scenarios under BS1; (D–F) The impact of the three integrated scenarios under BS2; (G–I) The impact of the three integrated scenarios under the HEC model. (BESS: battery energy storage system; PHS: pumped storage system).

The study further analyzes the contribution analysis of electricity carbon footprint under various SSPs. As shown in Figure 5, the contribution of solar power generation has changed significantly across scenarios with the same carbon footprint calculation method. Taking the scenarios with HEC model as an example, from right to left (from high carbon to low carbon), the proportion of solar power generation in the electricity carbon footprint is 0.34%, 4.45%, and 13.33% in year 2050. Meanwhile, different algorithms have a limited impact on the contribution of solar power generation to the carbon footprint of electricity (from top to bottom). For example, in the low-carbon scenario, the contribution of PV power generation to the carbon footprint of electricity is 13.33% (HEC model), 15.30% (BS1), and 7.75% (BS2), respectively. Therefore, no matter which method is used to calculate the carbon footprint, the decarbonization efforts can always be captured. But the HEC model can give a more robust projection.

This limited impact from carbon footprint calculation methods is also caused by the reliance of thermal power. As shown in Figure 5, even with the installation of CCS, the contribution of thermal power generation will continue to occupy a major fraction of the electricity mix carbon footprint, especially in medium and high-carbon scenarios. To meet the electricity demand and balance the intermittency of renewable energy, thermal power generation will continue to play a crucial role, ensuring a stable, and reliable energy supply.

#### Temporal legacy analysis of electricity carbon footprint

**Figure 6:**
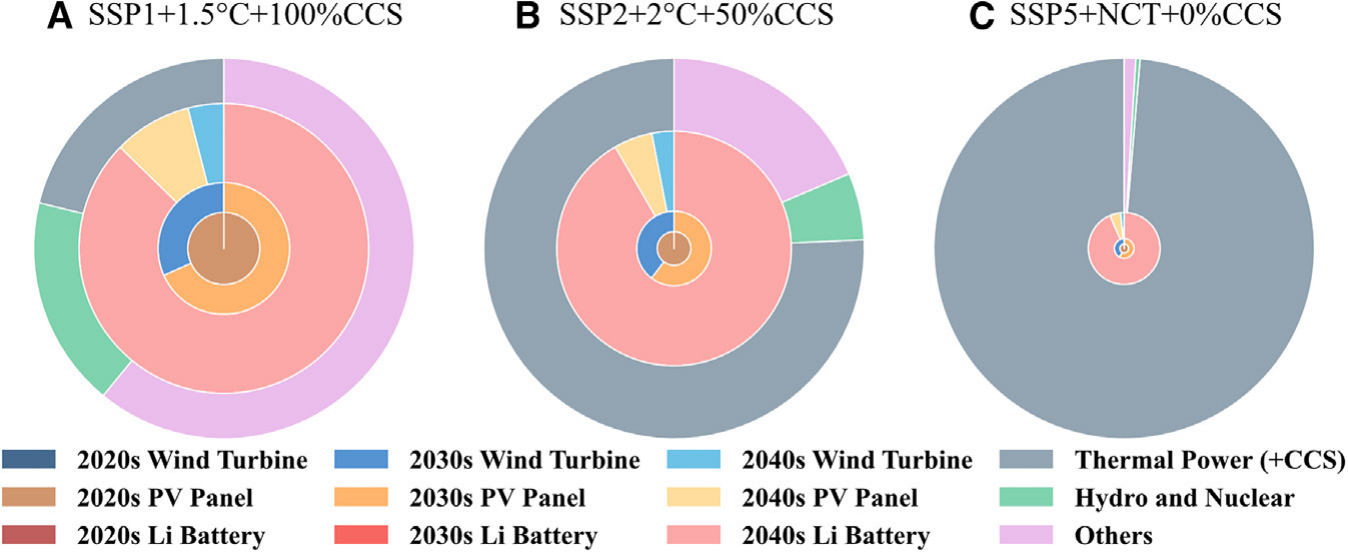
Projected carbon footprint contributions per kilowatt-hour of electricity in 2050 This is depicted under three distinct decarbonization pathways: (A) SSP1+1.5°C+100%CCS; (B) SSP2+2°C+50%CCS; (C) SSP5+NCT+0%CCS, delineating the sources of direct GHG emissions from thermal power with (or without) CCS and others, alongside indirect GHG emissions from the LCPI established in previous decades. The area of the ring indicates the contribution to the total carbon footprint across different decades.

Figure 6 sharply delineates the respective contributions of direct emissions from thermal power generation and the accumulated impact of low-carbon electricity infrastructure production on the carbon footprint of electricity by 2050. Within the SSP1+1.5°C+100%CCS framework—the most aggressive decarbonization pathway—a notable 4.49%, 13.33%, and 40.12% of the carbon footprint can be traced to the indirect GHG emissions from the solar, wind, and battery systems installed in past decades. This scenario highlights the enduring influence of GHG emissions from these previously installed LCPI on future emissions profiles, despite a deliberate move toward greener practices.

**Figure 7:**
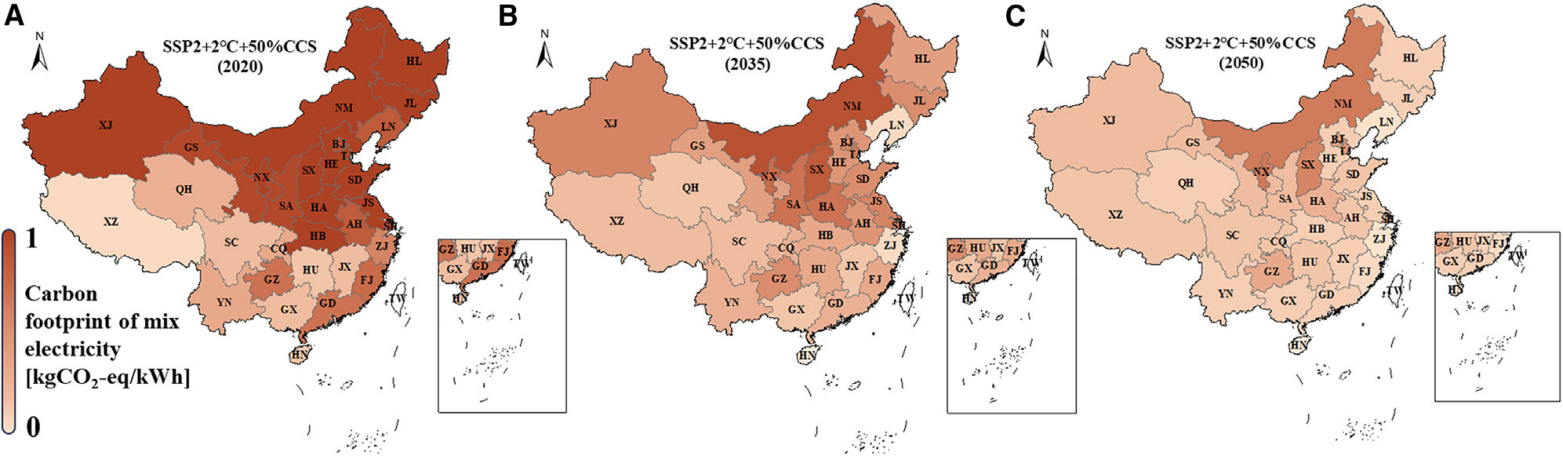
Spatiotemporal comparison of provincial electricity carbon footprint The spatiotemporal comparison of the carbon footprint of the provincial electricity mix in 2020 (A), 2035 (B), and 2050 (C). The background color of each province quantitatively represents the average carbon footprint of the provincial electricity mix. The missing provinces, namely Macau, Hong kong, and Taiwan, were not considered in this study. The spatiotemporal comparison of the carbon footprint of all in-service LCPI can be found in Figures S2 and S3. (see excel file Table S15 in supplemental information).

The intermediate SSP2+2°C+50%CCS scenario reveals a shared 37.99% contribution from both past renewable energy deployments and current thermal power operations, reflecting an insufficient reduction in carbon contributions from the electricity sector. Moreover, the thermal power’s carbon footprint increases markedly from 8.89% in the SSP1+1.5°C+100%CCS scenario to 46.96% in the SSP2+2°C+50%CCS scenario, accentuating the pivotal role of integrating CCS at higher percentages. In contrast, the SSP5+NCT+0%CCS scenario exhibits a dominant 95.14% carbon footprint from direct thermal power emissions, manifesting a continued heavy reliance on traditional, carbon-intensive electricity production with negligible renewable integration and no CCS adoption.

The contrasting scenarios clearly underscore the essentiality of prompt and sustained investment in low-carbon energy technologies to align future energy production with ambitious decarbonization goals. These quantified insights serve as a powerful call to action for policymakers, advocating for proactive energy planning that significantly reduces the long-term carbon footprint of electricity generation.

### Spatial heterogeneity analysis of electricity carbon footprint

Earlier discussions have juxtaposed the proposed HEC model with alternative methods, showcasing its distinct ability to capture synergistic decarbonization between the grid and LCPI. In this section, our attention shifts exclusively to the proposed HEC model, harnessing its elevated temporal-spatial resolution. Our aim is to offer practical and direct guidance for manufacturing planning, specifically in response to the implications of product carbon footprint management.

The high temporal-spatial resolution unveils noteworthy spatial disparities in grid decarbonization at the provincial level, as illustrated in Figures 7, S2, and S3. The chosen intermediate scenario, SSP2+2°C+50%CCS, accentuates these variations. Broadly speaking, the carbon footprint in northern regions markedly surpasses that in the southern regions. As the decarbonization unfolds, this disparity gradually diminishes, yet the overarching pattern of northern regions exhibiting higher carbon footprints than the southern regions remain relatively constant.

Given that the product carbon footprint is heavily influenced by the electricity footprint, this analysis serves as crucial guidance for manufacturers in their capacity expansion planning—essentially, determining optimal locations for constructing manufacturing plants. Unlike assessing local renewable capacity, the examination of electricity carbon footprint offers a nuanced perspective. For example, Qinghai province boasts abundant renewable resources and leads in solar and wind generation nationally. Despite this, the carbon footprint remains high in the first decade (2020–2030) due to LCPIs embedded with substantial GHG emissions. On the contrary, southern regions like Sichuan province exhibit limited solar and wind resources, yet the long-standing role of hydropower in local generation results in a notably low carbon footprint.

Consequently, the model suggests that, despite the rapid advancement of solar and wind technologies in northern regions, manufacturers aiming to swiftly reduce product footprints in the short term should contemplate relocating manufacturing capacity to provinces abundant in hydropower resources. Aligning with solar and wind development becomes more effective for lowering product carbon footprints in the medium and long term. When LCPIs themselves undergo sufficient decarbonization, they can subsequently reduce the carbon footprint of local electricity, leading to the low carbon footprint of local products.

## Discussion

This study enhances the importance of considering the accumulation effect of LCPI with various carbon footprints by HEC dynamic LCA model. Extending its application to explore the spatiotemporal heterogeneity of China’s electricity carbon footprints across various SSPs, carbon reduction targets, and CCS installation ratios. The developed framework provides a comprehensive approach to project the carbon footprint of electricity, a pivotal element in the calculation of carbon footprints for all products. It also guides coordinated decarbonization across the value chain of renewable energy industries. These insights are vital for developing policies and strategies that align with global climate goals and national commitments to a sustainable, low-carbon future.

Our study primarily focuses on the synergies between electricity decarbonization and the contributions of various renewable energy technologies. These results also have many meaningful implications.

The first policy implication is to facilitate the target setting from a life-cycle perspective. Previous research has shown that proposing the nationally determined contributions, a national climate target itself could be an effective measure in decarbonization.^39^ The quantitative results of electricity carbon footprint from this study can also serve as a baseline for companies and governments to evaluate their decarbonization efforts, fostering the transition of the energy industry and society toward a low-carbon future. These include minimizing process emissions and enhancing resource recycling, which are essential for complementing the substantial decarbonization besides the power sector.

Another key policy implication is to guide the budget planning and even capacity expansion planning of companies, particularly export-oriented countries and companies. For example, steel manufacturers can evaluate the carbon footprint composition of their exported products and estimate potential compliance cost savings by reallocating production to regions with lower electricity carbon footprints.^40^ They could also adopt electrification technologies, such as electric arc furnaces, to achieve deeper decarbonization by replacing fossil-based feedstocks.^41^^,^^42^^,^^43^ Our approach includes a long-term projection of carbon footprints with high spatiotemporal resolution, which can inform discounted net present value analyses for such decarbonization pathway. Although this study does not explicitly include electrification scenarios, it has accounted for electricity decarbonization in raw material production. In future work, we aim to expand this approach to cover a wider range of industries and detailed electrification pathways, further improving the model’s accuracy and predictive capability.

Despite the recent shift from organizational emission management to carbon footprint management in many policy settings, the national climate targets and related modeling tools like integrated assessment models (IAMs) are still emission based rather than footprint based. We would argue that if the IAM and related climate target is comprehensive enough, for example at global scale rather regional scale and at long term not temporary target, these modeling tools and policies are sufficient and effective. However, if the tools are used to facilitate the regional and temporary climate policy, we would highly recommend carbon footprint based on tools to prevent carbon leakage in both spatial and temporal perspectives.

Using China as a case study, this analysis integrates various resource endowments in different provinces with diverse development scenarios. The insights derived from this approach are transferable to many regions globally, as the varied development scenarios and resource endowments in different provinces provide a broad spectrum adaptable to local conditions. From a methodological perspective, the framework proposed in this study can be applied to other regions, offering a detailed guide for product carbon footprint management.

Beyond carbon footprint analysis, the proposed model can be expanded to assess the comprehensive environmental footprints of products in the context of future grid decarbonization. For example, the reduction of atmospheric pollution from traditional thermal power generation can be included as part of the renewable energy transition.^44^ Additionally, the extended model facilitates a systematic evaluation of pollutants and the critical metal demand associated with the life cycle of LCPI.^45^^,^^46^ Ultimately, this approach enables manufacturers to enhance sustainable product design and life cycle management strategies under various energy transition scenarios.

### Limitations of the study

Finally, this study has certain limitations that warrant attention in future research. (1) The reliance on a proxy group of renewable technologies in the projections, without accounting for the burgeoning fields of offshore wind, concentrated solar power, and various long-duration energy storage technologies, introduces a bias. (2) The study assumes a uniform theoretical lifespan for all technologies, neglecting variations in lifespan due to regional and use-case factors. For instance, batteries in colder regions may have shorter lifespans compared to those in warmer regions.^47^^,^^48^ (3) The analysis also lacks a rigorous consideration of waste management for energy infrastructure, potentially underestimating the system’s overall carbon footprint. Recycling introduces unique flows between retired components and in-use inventory at the product level, which could lead to synergistic and legacy effects on the carbon footprint. (4) The holistic assessment of grid decarbonization potential has been limited by the lack of thorough consideration of other factors such as the structure of freight fuel, bio-power generation technology, and the construction of electrical infrastructure (e.g., underground cables, transformers, substation equipment). Furthermore, transmission losses and emissions of sulfur hexafluoride (SF_6_) from the grid have not been fully accounted for. These aspects require further in-depth exploration to obtain a more comprehensive understanding of the grid’s carbon footprint.^49^ A sensitivity analysis was conducted on the gradual decarbonization of components other than electricity and its impact on the carbon footprint, with detailed content available in Section S4 with Table S5 and Figure S4 of supplemental information. (5) The data in this paper mainly comes from Ecoinvent V3.7, and it is true that there are issues with outdated data. Additionally, due to the lack of a fully developed and widely recognized energy decarbonization model, the forecast of electricity demand relies on assumptions from original studies, which could introduce some degree of bias. However, the main purpose of this article is to propose a new model to explore the potential for synergistic decarbonization of LCPI and energy transition. By conducting a comprehensive analysis of various scenarios, efforts have been made to overcome data bias issues and enhance the reliability of the results. Future work could involve developing more detailed models to further refine electricity demand forecasts. This limitation has been acknowledged in the article to encourage future improvements. (6) This paper establishes a relatively simplified CCS carbon footprint model using global data. Given the development prospects of CCS technology, it is essential to incorporate a full life cycle analysis of CCS technology (including GHG emissions from equipment manufacturing, installation, and operation phases) into the model in the future. It is also crucial to fully demonstrate how energy use and material demand change as the scale of CCS technology expands. (7) The study only considers the life cycle GHG emission differences of certain power generation methods across provinces. Future work should improve data and model to consider carbon footprint differences caused by the inter provincial material trade, to deeply understand the spatial differences in China’s electricity carbon footprint. In summary, although there are these issues, they do not detract from the main contributions of this research. Instead, they highlight future areas for exploration based on the results of this study.

## Resource availability

### Lead contact

Further information and requests for resources and code should be directed to and will be fulfilled by the lead contact, Jiaqi Lu (wilsherelu@foxmail.com).

### Materials availability

This study did not generate new unique reagents.

### Data and code availability

• Data reported in this article can be found in the supporting information.
• All original code for the modeling has been deposited at Zenodo and publicly available as of the date of publication. DOIs are listed in the key resources table.
• Any additional information reported in this study is available from the lead contact upon request.

## Acknowledgments

This work was supported by 10.13039/501100001809National Natural Science Foundation of China (no.52400242) and Capacity Building Project of Local Colleges and Universities in Shanghai (no.21010501400) from 10.13039/501100003399Science and Technology Commission of Shanghai Municipality.

## Author contributions

J.T.: methodology, software, data curation, writing—original draft. R.S.: writing—original draft, conceptualization, writing—review and editing. P.W.: data support, conceptualization. W.-Q.C.: data support, conceptualization. D.G.: investigation, writing—review and editing. G.L.: conceptualization, writing—review and editing. P.R.: funding acquisition, project administration. J.W.: conceptualization, writing—review and editing. J.L.: conceptualization, supervision, writing—review and editing.

## Declaration of interests

The authors declare no competing interests.

## STAR★Methods

### Key resources table

**Table:** 

REAGENT or RESOURCE	SOURCE	IDENTIFIER
**Deposited data**		

Original code	This paper	https://doi.org/10.5281/zenodo.14632414

**Software and algorithms**		

OpenLCA 1.10.3	GreenDelta GmbH	https://www.openlca.org/
MATLAB R2017a	MathWorks	https://www.mathworks.com/products/matlab.html
IPCC GWP 100a	openLCA Nexus	https://nexus.openlca.org/database/openLCA%20LCIA%20methods

**Other**		

ecoinvent database V3.7	ecoinvent	https://ecoinvent.org/

### Method details

This study extends the previously proposed HEC dynamic LCA model^27^ by incorporating comprehensive renewable energy-driven power systems, including PV panels, wind turbines, and energy storage systems. The model integrates a provincial energy transition framework and captures the dynamic decarbonization and accumulation of annually manufactured LCPI. It also considers critical factors such as socio-economic development, climate target, and CCS deployment. This enhanced model enables precise quantification of the historical cumulative contribution and spatiotemporal heterogeneity of LCPI manufacturing under various energy transition scenarios. By recording the annual installation of PV panels, wind turbines, and battery components with varying carbon footprints based on their production year, the model deciphers power grid decarbonization trajectories and provides essential insights into historical emission responsibility, supporting the achievement of carbon neutrality.

#### The system boundary of the HEC dynamic LCA framework

In this study, the functional unit is defined as the carbon footprint of producing 1 kWh of electricity by China's provincial grids from 2020 to 2050. As shown in Figure 1, the system boundaries include the cradle-to-gate power consumption and upstream material consumption for the production of photovoltaic (PV) panels, wind turbines, and lithium batteries.^50^ Along with the installed power infrastructures, the power grid also includes thermal power plants (with CCS), biomass (with CCS),^51^ nuclear, hydroelectric, and pumped hydro storage. The temporal resolution of this model is set at an annual timestep, taking into account the decarbonization of both newly produced LCPI and cumulative installations. This implies that the carbon footprint of the power grid incorporates the embodied GHG emissions from LCPI produced in different years, each characterized by varying product carbon footprints. The geographic heterogeneity of the model considers the provincial difference in the energy structure. It is assumed that the carbon footprint of electricity derived from sources other than LCPI remains constant throughout the study period. The model does not include the post-consumer waste treatment and recycling of LCPI. Additionally, the gradual performance degradation of components over time is not considered in the model.

#### Model description

Based on the defined system boundaries, this section describes the method for quantifying the carbon footprint of provincial-level electricity generation. The LCPI is divided into variable renewable energy (VRE)^52^ including wind and solar energy, and Battery Energy Storage (BES).^53^ The annual carbon footprint of newly installed VRE (*ECF*_VER-new_) and BES (*ECF*_BES-new_) components is calculated by dividing the GHG emissions from producing 1 kWp (kilo Watt peak, denoting the peak total power of photovoltaic solar cells) of solar panels, 1 MW of wind turbines, and 1 kg of lithium batteries from raw material extraction to infrastructure manufacture (i.e., cradle to gate) into electricity consumption processes (*GHG*_ele_) and other processes (*GHG*_other_).^27^ The total electricity contribution is the electricity consumption of the LCPI multiplied by the annual national average electricity carbon footprint (*ECF*_nat_).

(Equation 1) $$\begin{array}{l} GHG\left(p,t\right)={GHG}_{\text{ele}}\left(p,t\right)+{GHG}_{\text{other}} \end{array}$$

(Equation 2) $$\begin{array}{l} {GHG}_{\text{ele}}\left(p,t\right)=E_{\text{LCPI}}\times {ECF}_{\text{na}t} \end{array}$$

where the electricity carbon footprint for calculating *GHG*_ele_ is influenced by the province (*p*) and time (*t*) of LCPI production; while, other carbon footprint (*GHG*_other_) from heat consumption, raw material extraction, etc., is set as a constant.

The cradle-to-gate GHG emissions from annually installed LCPI during production are then allocated to the total electricity generation or storage capacity throughout their life cycle.

(Equation 3) $$\begin{array}{l} {ECF}_{\text{VRE}-\text{new}}\left(p,t,n\right)=\frac{GHG\left(p,t,n\right)}{{AE}_{\text{out}}\times LS} \end{array}$$

(Equation 4) $$\begin{array}{l} {ECF}_{\text{BES}-\text{new}}\left(p,t\right)=\frac{{GHG}_{\text{BES}}\left(p,t\right)}{ED\times LS} \end{array}$$

Here, *AE*_out_ represents the annual electricity output per kWp of solar panels or per MW of wind turbines in each province, determined by solar or wind energy resources such as the available electricity output period in a year, and; *ED* is the average energy density of lithium-ion batteries, valued at 160 Wh/kg^54^; *LS* [yr] is the component's lifespan, with PV panels set at 25 years,^55^ wind turbines at 20 years,^56^ and lithium batteries at 10 years.^54^ Wind and solar components are denoted by n=1 and n=2, respectively. For PHS, only the energy storage carbon footprint of the operating process (*ECF*_PHS-new_) is considered in this paper.

The annual carbon footprint of wind and solar components in service within the grid (*ECF*_VRE-avg_) is calculated as a weighted average based on their cumulative electricity generation. However, as lithium battery storage and Pumped Hydro Storage do not belong to electricity generation facilities, the carbon footprint of energy storage systems (*ECF*_ESS-avg_) is determined by dividing the annually allocated carbon footprint of installed LBS and PHS storage with the total electricity generation of that year (*ELE*_sum_).

(Equation 5) $$\begin{array}{l} {ECF}_{\text{VER}-\text{avg}}\left(p,t,n\right)=\frac{\underset{i=t-LS+1}{\overset{t}{\sum }}{ECF}_{\text{VER}-\text{new}}\left(p,i,n\right)\times {ELE}_{\text{VER}-\text{new}}\left(p,i,n\right)}{\underset{i=t-LS+1}{\overset{t}{\sum }}{ELE}_{\text{VER}-\text{new}}\left(p,i,n\right)} \end{array}$$

(Equation 6) $$\begin{array}{l} {ECF}_{\text{ESS}-\text{avg}}\left(p,t,l\right)=\frac{\underset{i=t-LS+1}{\overset{t}{\sum }}{ECF}_{\text{ESS}-\text{new}}\left(p,i,l\right)\times {ELE}_{\text{ESS}-\text{new}}\left(p,i,l\right)}{{ELE}_{\text{sum}}\left(p,i\right)} \end{array}$$

In the formula, *ELE*_VRE-new_ and *ELE*_ESS-new_ [kWh] represent the annual electricity generation and storage capacity of newly installed LCPI added by VRE components and ESS respectively in a given year. The variable ‘*i*' is used to differentiate the production year of all components used in the ‘*t-th*' year; ‘*l*' represents LBS and PHS.

From the above equations, it is possible to derive the provincial-level electricity mix carbon footprint (*ECF*_pro_ [kg CO_2_-eq/kWh]) and the national average electricity carbon footprint (*ECF*_nat_ [kg CO_2_-eq/kWh]):

(Equation 7) $$\begin{array}{l} {ECF}_{\text{pro}}\left(p,t\right)=\\ \frac{\sum_{k}\;{ECF}_{\text{other}}\left(k\right)\times {ELE}_{\text{other}}\left(p,t,k\right)+\underset{i=t-LS+1}{\sum^{t}}{ECF}_{\text{VER}-\text{new}}\left(p,i,n\right)\times {ELE}_{\text{VER}-\text{new}}\left(p,i,n\right)}{\sum_{k}{ELE}_{\text{other}}\left(p,t,k\right)+{\sum_{n}\underset{i=t-LS+1}{\sum^{t}}ELE}_{\text{VER}-\text{new}}\left(p,i,n\right)}+\sum_{l}{ECF}_{ESS-avg}\left(p,t,l\right) \end{array}$$

(Equation 8) $${ECF}_{\text{nat}}\left(t\right)=\frac{\sum_{p}({ECF}_{\text{pro}}\left(p,t\right)\times (\sum_{k}{ELE}_{\text{other}}\left(p,t,k\right)+\sum_{n}\sum_{i=t-LS+1}^{t}{ELE}_{\text{LCPI}-\text{new}}\left(p,i,n\right)))}{\sum_{p}\left(\sum_{k}{ELE}_{\text{other}}\left(p,t,k\right)+\sum_{n}\sum_{i=t-LS+1}^{t}{ELE}_{\text{LCPI}-\text{new}}\left(p,i,n\right)\right)}+\frac{\sum_{p}\left(\sum_{l}{ECF}_{ESS-avg}\left(p,t,l\right)\times {ELE}_{\text{ESS}-\text{new}}\left(p,i,l\right)\right)}{\sum_{p}{ELE}_{\text{sum}}\left(p,i\right)}$$

*ECF*_other_ [kg CO_2_-eq/kWh] represents the carbon footprint (*k*) of eight different electricity sources, including coal, natural gas, biomass, hydropower, nuclear power, and electricity generated from thermal and biomass integrated with CCS technologies. *ELE*_other_[kWh] denotes the corresponding electricity generation amount for each of these energy sources.

In 2020, a portion of VRE has already installed in the grid. However, in the initial year, there were issues with unclear production timelines and missing carbon footprint data for these components. According to reports from the National Energy Administration, photovoltaic power generation began to be deployed extensively in 2011, and wind power saw large-scale installations from 2016 onwards.^57^^,^^58^ Consequently, in the metabolism scenario, it is assumed that all operational photovoltaic panels in 2020 will be uniformly replaced by new panels between 2036-2045, and wind turbines will be gradually replaced between 2036-2040, with newly added components in subsequent years being completely replaced at the end of their lifespan. Lithium-ion battery energy storage will not begin to scale until 2025, so 2025 will be used as the initial year for its carbon footprint calculation.^59^

Finally, *ELE*_VRE-new_ and *ELE*_BES-new_ in different years can be determined by a piecewise function of the cumulative electricity generation (*EG*_sum_ [TWh]) and electricity stored (*ES*_sum_ [TWh]) of the in-service infrastructures, and the formula is detailed in supplemental information.

#### The cumulative effects of the LCPI production process compared with benchmarks

The national average of electricity carbon footprint (*ECF*_nat_) affects the GHG emissions due to the cradle-to-gate electricity consumption for LCPI manufacture (*GHG*_ele_). Further, the exponential increase of LCPI installation contributes to the energy structure transition towards grid decarbonization. Taking into account that the annual electricity production comes from both the LCPI produced in the current year and the LCPI produced in previous years, which have different carbon footprints, this model calculates the historical cumulative impact of carbon in the LCPI production process on the power grid. In order to quantify the synergistic decarbonization behavior between LCPI manufacturing and the power grid under the cumulative effect of heterogenous-embodied-carbon devices, we have defined two comparison benchmarks for validating the proposed model. As shown in Table 1, the first benchmark (BS1) is based on the constant *GHG*_ele_ as reported in Ecoinvent V3.7, where results are only influenced by the energy structure of the power grid. This benchmark illustrates an extreme situation in which the future LCPI industry chain will not decarbonize along with the power grid, thereby allowing for a comparative analysis to investigate whether the decarbonization of LCPI has a significant influence on electricity carbon footprint. The second benchmark (BS2) considers the decarbonization of LCPI manufacture without accounting for the heterogenous carbon footprint decided by the production year, reflecting the algorithm in the previous literature.^25^^,^^60^ Thus, the carbon footprint of LCPI will change based on the electricity carbon footprint in the corresponding year, without the information of historical production records.

#### Scenario analysis

To facilitate a better understanding of the HEC dynamic LCA model, we considered different temperature targets, varying CCS installation rates, and SSP scenarios. This approach should capture the scenario of dual carbon goal proposed by Chinese government.^61^ The Intergovernmental Panel on Climate Change (IPCC) introduced five socioeconomic transformation pathways in 2010,^62^^,^^63^^,^^64^^,^^65^ namely SSP1 (Sustainability Pathway), SSP2 (Middle-of-the-Road Pathway), SSP3 (Regional Competition Pathway), SSP4 (Inequality Pathway), and SSP5, which is similar to SSP3 but more reliant on traditional fossil fuels. These pathways represent low, medium, and high challenges for both mitigation and adaptation, with SSP1, SSP2, and SSP3 representing low, medium, and high challenges for adaptation and mitigation.^66^ (Table S1 provides a qualitative description of the SSPs' development paths for future socioeconomic and electricity sector trends. In order to balance the power grid system, the mandatory retirement policy of coal power is not considered.).

Additionally, as shown in the report 'Pathways to deep decarbonization in China' by the National Center for Climate Change Strategy and International Cooperation (NCSC), Tsinghua University, and other institutes, the carbon dioxide intensity of electricity in 2050 is projected to be 68 g/kWh under the 2°C target. The ' Special Report on Global Warming of 1.5°C' indicates that under the 1.5°C target, the power sector is expected to achieve carbon neutrality by 2050.^67^^,^^68^ Therefore, in order to identify the impacts of different temperature control targets, we have model scenarios including a no climate targets scenario (NCT) without emission reduction constraints, a deep decarbonization target (2°C),^69^ and a net-zero emission target (1.5°C).^36^ The contribution of CCS technology to emission reductions in China's energy and power sector is expected to increase gradually with the overall demand for electricity and the acceleration of the low-carbon transition.^70^ Therefore, we explore the impact of thermal power generation with varying proportions of CCS on the carbon footprint of the power grid (Table 1). In this model, the introduction of the CCS assumption is based on a linear increase by varying the penetration ratio in 2050. For the high uncertainty of CCS deployment, it is hard to integrate the CCS scenarios with SSPs and climate target. 0-100% of the penetration ratio in 2050 is fully investigated to identify the impact of CCS for calculating the carbon footprint of electricity.

#### Data sources

Data for the production of 1kWp ground-mounted solar panels, 1MW onshore wind turbines, and 1kg lithium-ion batteries were converted from inventory data in Ecoinvent V3.7 (Table S6 in supplemental information).^28^ The corresponding greenhouse gas emissions were calculated using the IPCC 2013 GWP 100a method (Global Warming Potential). CCS, a high-cost but essential carbon-neutral technology, is expected to be extensively developed in the future; while the simulated installation is limited in China. Based on the life cycle assessment work dataset of Volkart and colleagues on carbon capture and storage for power generation and industry in Europe,^37^ we establish an operational phase model with Ecoinvent V3.7 that can be representative of CCS technology in China. The reference inventory data are provided in Tables S7 and S8 in supplemental information.

The provincial-level electricity production structure prediction model, constructed by Li's team (the excel file Table S12 in supplemental information),^29^ provided the cumulative installed capacity and generated electricity of all power sources in China's 31 provinces from 2020 to 2050, under different GHG emission reduction targets following SSPs development pathways. As energy storage systems are complementary technologies to unstable renewable energy, their installed capacities should be closely dependent on the penetration ratio of hydro, solar, and wind power in the grid. We sourced the cumulative installed capacity of all power sources for a given state and year in the United States from the standard scenario reports (mid-term scenario) modeled by the National Renewable Energy Laboratory.^71^ Using this data as a statistical sample, we established a linear model relating the installed capacities of energy storage systems to those of renewable energy, which was assumed as a universal estimation method for predicting the energy storage capacity in a power grid dominated by unstable energy, and to estimate the total storage capacity of each province in China (Tables S9 and S10 in supplemental information). The total storage capacity is then allocated by the ratio of pumped storage and lithium battery energy storage estimated by Zhuo^52^ in each province of China, and finally, the pumped storage and battery storage capacity of each province in China is obtained. See supplemental information for specific expressions and data.

The annual electricity generation per kWp PV panels of each province in China came from the Global Solar Atlas 2.0 (Solargis on behalf of World Bank Group, 2022).^72^ In the Global Wind Energy Atlas 3.0 (Windgis is the product of a partnership between the World Bank Group and the Department of Wind Energy at the Technical University of Denmark),^73^ five points (1000 km^2^) with the strongest wind density in each province were selected. The data was extracted using Matlab software, and the average value was calculated, and finally, the annual wind power generation per MW of each province was obtained. (Table S11 in supplemental information).
