# Supplementary material for: Deciphering decarbonization trajectories in China by spatiotemporal-accumulation modeling of electricity carbon footprint
Source: iScience. 2025 Feb 6;28(3):111963. doi: 10.1016/j.isci.2025.111963 (PMC11907481; doi:10.1016/j.isci.2025.111963)
Supplement: Document S1. Figures S1–S4 and Tables S1–S11 [file mmc1.pdf]

**iScience, Volume *28***

## Supplemental information

# Deciphering decarbonization trajectories in China by spatiotemporal-accumulation modeling of electricity carbon footprint

**Jing Tang, Rui Shan, Peng Wang, Wei-Qiang Chen, Dungang Gu, Guanghui Li, Pinhua Rao, Jinguo Wang, and Jiaqi Lu**

*Table S1. Qualitative description of Shared Socioeconomic Pathways related to Introduction.[1,2] Related to INTRODUCTION.*

<table>
<tr><th></th><th>SSP1</th><th>SSP2</th><th>SSP3</th><th colspan="2">SSP4</th><th>SSP5</th></tr>
<tr><td>GDP</td><td>Middle</td><td>Middle</td><td>High</td><td colspan="2">Middle</td><td>High</td></tr>
<tr><td>Population</td><td>Low</td><td>Middle</td><td>High</td><td colspan="2">Low</td><td>High</td></tr>
<tr><td>Technological progress</td><td>Middle</td><td>Middle</td><td>Low</td><td colspan="2">High</td><td>High</td></tr>
<tr><td>Fossil fuel supply</td><td>Middle</td><td>Middle</td><td>High</td><td>Low</td><td>High</td><td>High</td></tr>
<tr><td>Power demand intensity</td><td>Low</td><td>Middle</td><td>Middle</td><td>Low</td><td>Middle</td><td>High</td></tr>
</table>

*Table S2. Abbreviations of provinces in Chinese Mainland. Related to INTRODUCTION.*

| Province name | Abbreviation | Province name | Abbreviation |
|---|---|---|---|
| Anhui | AH | Jiangxi | JX |
| Beijing | BJ | Liaoning | LN |
| Chongqing | CQ | Inner Mongolia | NM |
| Fujian | FJ | Ningxia | NX |
| Guangdong | GD | Qinghai | QH |
| Gansu | GS | Shaanxi | SA |
| Guangxi | GX | Sichuan | SC |
| Guizhou | GZ | Shandong | SD |
| Henan | HA | Shanghai | SH |
| Hubei | HB | Shanxi | SX |
| Hebei | HE | Tianjin | TJ |
| Heilongjiang | HL | Xinjiang | XJ |
| Hanan | HN | Tibet | XZ |
| Hunan | HU | Yunnan | YN |
| Jilin | JL | Zhejiang | ZJ |
| Jiangsu | JS | | |

Due to a lack of data, this article did not consider Taiwan, Hong Kong, and Macau.

*Table S3. Division of China's Four Major Geographical Regions. Related to INTRODUCTION.*

| Geographical division | Abbreviation |
|---|---|
| Northern region | BJ, HE, HA, HL, JL, LN, SD, SX, TJ |
| Southern region | AH, FJ, GD, GX, GZ, HN, HB, HU, JS, JX, SH, ZJ, CQ， MO, HK, TW |
| Northwest region | SA, GS, QH, NX, XJ |
| Qinghai Tibet region | XZ, QH, SC, YN |

*Table S4. Changes in the carbon footprint of battery storage over time under baseline scenario 1(BS1), baseline scenario 2(BS2), and the HEC model scenarios (Model). Related to INTRODUCTION.*

| Year | BS1[kg$CO_2$-eq/kWh] | BS2[kg$CO_2$-eq/kWh] | Model[kg$CO_2$-eq/kWh] |
|---|---|---|---|
| 2025 | 0.06656 | 0.06642 | 0.06646 |
| 2026 | 0.06876 | 0.06854 | 0.06859 |
| 2027 | 0.07096 | 0.07064 | 0.07069 |
| 2028 | 0.07317 | 0.07272 | 0.07278 |
| 2029 | 0.07539 | 0.07479 | 0.07485 |
| 2030 | 0.07762 | 0.07684 | 0.07690 |
| 2031 | 0.08381 | 0.08255 | 0.08262 |
| 2032 | 0.09021 | 0.08832 | 0.08840 |
| 2033 | 0.09684 | 0.09416 | 0.09425 |
| 2034 | 0.10367 | 0.10002 | 0.10013 |
| 2035 | 0.04015 | 0.03592 | 0.03599 |
| 2036 | 0.04921 | 0.04272 | 0.04283 |
| 2037 | 0.05556 | 0.04679 | 0.04693 |
| 2038 | 0.06185 | 0.05052 | 0.05070 |
| 2039 | 0.06808 | 0.05392 | 0.05414 |
| 2040 | 0.07425 | 0.05700 | 0.05727 |
| 2041 | 0.07957 | 0.05826 | 0.05858 |
| 2042 | 0.08479 | 0.05922 | 0.05960 |
| 2043 | 0.08991 | 0.05991 | 0.06034 |
| 2044 | 0.09495 | 0.06036 | 0.06084 |
| 2045 | 0.09648 | 0.05720 | 0.06114 |
| 2046 | 0.09729 | 0.05433 | 0.05841 |
| 2047 | 0.09809 | 0.05172 | 0.05597 |
| 2048 | 0.09888 | 0.04923 | 0.05362 |
| 2049 | 0.09965 | 0.04684 | 0.05138 |
| 2050 | 0.10042 | 0.04456 | 0.04924 |

*Table S5. Scenario design for sensitivity analysis the other carbon footprint contribution. Related to DISCUSSION.*

| Scenario | Brief Description |
|---|---|
| Baseline | $ECF_{nat}$ only changes with the change of power structure |
| $GHG_{ele}$ | $ECF_{nat}$ changes with the decarbonization of the electricity sector in LCPI. |
| 25% $GHG_{other}$ | Assuming that by 2050, the other parts of LCPI will decrease by 25% based on 2020 levels, with the remaining years decreasing linearly. $ECF_{nat}$ changes as LCPI's electricity and other parts decarbonize. |
| 50% $GHG_{other}$ | Assuming that by 2050, the other parts of LCPI will decrease by 50% based on 2020 levels, with the remaining years decreasing linearly. $ECF_{nat}$ changes as LCPI's electricity and other parts decarbonize. |
| 75% $GHG_{other}$ | Assuming that by 2050, the other parts of LCPI will decrease by 75% based on 2020 levels, with the remaining years decreasing linearly. $ECF_{nat}$ changes as LCPI's electricity and other parts decarbonize. |
| 100% $GHG_{other}$ | Assuming that by 2050, the other parts of LCPI will decrease by 100% based on 2020 levels, with the remaining years decreasing linearly. $ECF_{nat}$ changes as LCPI's electricity and other parts decarbonize. |

* $ECF_{nat}$: the national average electricity carbon footprint; $GHG_{ele}$: Emissions from LCPI electricity consumption; $GHG_{other}$: Emissions generated by parts of LCPI other than electricity consumption.

*Table S6. Referred database about the electricity generation by different energy sources. Abbreviation means the located power grid of each province.*[3] *Related to STAR★Methods.*

| Energy sources | Database |
|---|---|
| Coal | Ecoinvent: electricity production, hard coal \| electricity, high voltage \| Cutoff, U-CN- *Abbreviation* |
| Natural gas combined cycle | Ecoinvent: electricity production, natural gas, combined cycle power plant \| electricity, high voltage \| Cutoff, U-CN- *Abbreviation* |
| Natural gas combustion turbine | Ecoinvent: electricity production, natural gas, conventional power plant \| electricity, high voltage \| Cutoff, U-CN- *Abbreviation* |
| Biogas | Ecoinvent: heat and power co-generation, biogas, gas engine \| electricity, high voltage \| Cutoff, U-RoW |
| Hydro | Ecoinvent: electricity production, hydro, reservoir, non-alpine region \| electricity, high voltage \| Cutoff, U-RoW<br><br>Ecoinvent: electricity production, hydro, run-of-river \| electricity, high voltage \| Cutoff, U- *Abbreviation* |

| | |
|---|---|
| Nuclear power | Ecoinvent: electricity production, nuclear, boiling water reactor \| electricity, high voltage \| Cutoff, U-RoW<br><br>Ecoinvent: electricity production, nuclear, pressure water reactor \| electricity, high voltage \| Cutoff, U- *Abbreviation* |
| Pumped hydro storage | Ecoinvent: electricity production, hydro, pumped storage \| electricity, high voltage \| Cutoff, U-CN- *Abbreviation* |
| Wind | Ecoinvent: market for wind turbine, 4.5MW, onshore \| wind turbine, 4.5MW, onshore \| Cutoff, U-Global |
| Photovoltaic | Ecoinvent: photovoltaic plant construction, 570kWp, multi-Si, on open ground \| photovoltaic plant, 570kWp, multi-Si, on open ground \| Cutoff, U-Global |
| Lithium battery | Ecoinvent: battery production, Li-ion, rechargeable, prismatic \| battery, Li-ion, rechargeable, prismatic \| Cutoff, U-Global |

*Table S7. Reference Database for CCS Technology based on Volkart and colleagues on carbon capture and storage for power generation and industry in Europe.[4] Related to STAR★ Methods.*

| Energy sources | Dataset |
|---|---|
| Coal-CCS | $CO_2$ capture/hard coal, post, pipeline 200km, storage 1000m |
| Gas-CCS | $CO_2$ capture/nature gas, post, pipeline 200km, storage 1000m |
| Biogas-CCS | $CO_2$ capture/at $H_2$ production, pre, pipeline 200km, storage 1000m |

*Table S8. Carbon footprint of power generation with CCS in each province. Related to STAR ★Methods.*

| Province | Biogas-CCS [kg$CO_2$-eq/kWh] | Coal-CCS [kg$CO_2$-eq/kWh] | Gas-CCS [kg$CO_2$-eq/kWh] |
|---|---|---|---|
| BJ | 0.17405 | 0.28870 | 0.18062 |
| CQ | 0.17405 | 0.40800 | 0.25167 |
| FJ | 0.17405 | 0.32168 | 0.19747 |
| GD | 0.17405 | 0.32112 | 0.19747 |
| GS | 0.17405 | 0.35942 | 0.22128 |
| GX | 0.17405 | 0.36047 | 0.22128 |
| GZ | 0.17405 | 0.36023 | 0.22128 |
| HA | 0.17405 | 0.34785 | 0.21481 |
| HB | 0.17405 | 0.38178 | 0.23550 |
| HE | 0.17405 | 0.37133 | 0.22818 |
| HL | 0.17405 | 0.45360 | 0.28061 |
| HN | 0.17405 | 0.35962 | 0.22128 |
| HU | 0.17405 | 0.34830 | 0.21481 |
| JL | 0.17405 | 0.47488 | 0.29179 |
| JS | 0.17405 | 0.31332 | 0.19230 |
| JX | 0.17405 | 0.34817 | 0.21481 |
| LN | 0.17405 | 0.40857 | 0.25167 |
| NM | 0.17405 | 0.51221 | 0.31707 |
| NX | 0.17405 | 0.39560 | 0.24332 |
| QH | 0.17405 | 0.39432 | 0.24332 |
| SA | 0.17405 | 0.34011 | 0.20870 |
| SC | 0.17405 | 0.38201 | 0.23550 |
| SD | 0.17405 | 0.35910 | 0.22128 |
| SH | 0.17405 | 0.29783 | 0.18274 |
| SX | 0.17405 | 0.39385 | 0.24332 |
| TJ | 0.17405 | 0.34920 | 0.21481 |
| XJ | 0.17405 | 0.37132 | 0.22818 |

| | | | |
|---|---|---|---|
| XZ | 0.17405 | 0.36534 | 0.22400 |
| YN | 0.17405 | 0.51396 | 0.31707 |
| ZJ | 0.17405 | 0.29796 | 0.18274 |

*Table S9. Proportion of Lithium Battery Energy Storage (LBES) and Pumped Storage (PHS) in Various Provinces of China based on the work content of Zhou et al.*[5] *Related to STAR★ Methods.*

| Province | LBES | PHS | Province | LBES | PHS |
|---|---|---|---|---|---|
| AH | 34.66% | 65.34% | JX | 49.97% | 50.03% |
| BJ | 6.90% | 93.10% | LN | 59.09% | 40.91% |
| CQ | 41.62% | 58.38% | NM | 33.73% | 66.27% |
| FJ | 54.22% | 45.78% | NX | 2.94% | 97.06% |
| GD | 10.84% | 89.16% | QH | 65.90% | 34.10% |
| GS | 8.99% | 91.01% | SA | 20.65% | 79.35% |
| GX | 12.25% | 87.75% | SC | 21.70% | 78.30% |
| GZ | 33.45% | 66.55% | SD | 22.46% | 77.54% |
| HA | 21.66% | 78.34% | SH | 0.00% | 100.00% |
| HB | 85.13% | 14.87% | SX | 23.33% | 76.67% |
| HE | 42.20% | 57.80% | TJ | 0.03% | 99.97% |
| HL | 86.45% | 13.55% | XJ | 18.66% | 81.34% |
| HN | 99.00% | 1.00% | XZ | 36.87% | 63.13% |
| HU | 19.61% | 80.38% | YN | 43.07% | 56.93% |
| JL | 63.94% | 36.06% | ZJ | 44.73% | 55.27% |
| JS | 18.86% | 81.14% | | | |

*Table S10. The linear fit ($R^2$), coefficient (K), and significance (F) based on the power grid structure data of 42 states in the United States from 2022 to 2050 as the statistical sample.[6] Related to STAR★Methods.*

| State | Abbreviation | $R^2$ | K | F |
|---|---|---|---|---|
| Alabama | AL | 0.793 | 0.890 | <0.001 |
| Arkansas | AR | 0.563 | 0.771 | <0.001 |
| Arizona | AZ | 0.841 | 0.923 | <0.001 |
| California | CA | 0.989 | 0.994 | <0.001 |
| Colorado | CO | 0.609 | 0.781 | <0.001 |
| Connecticut | CT | 0.822 | 0.907 | <0.001 |
| Delaware | DE | 0.414 | 0.675 | 0.006 |
| Florida | FL | 0.874 | 0.935 | <0.001 |
| Georgia | GA | 0.824 | 0.951 | <0.001 |
| Iowa | IA | 0.987 | 0.994 | <0.001 |
| Idaho | ID | 0.003 | -0.272 | 0.327 |
| Illinois | IL | 0.506 | 0.736 | 0.002 |
| Indiana | IN | 0.671 | 0.834 | <0.001 |
| Kansas | KS | 0.852 | 0.923 | <0.001 |
| Kentucky | KY | 0.819 | 0.912 | <0.001 |
| Louisiana | LA | 0.830 | 0.938 | <0.001 |
| Massachusetts | MA | 0.852 | 0.929 | <0.001 |
| Maryland | MD | 0.05 | 0.344 | 0.209 |
| Maine | ME | 0.772 | 0.888 | <0.001 |
| Michigan | MI | 0.935 | 0.970 | <0.001 |
| Minnesota | MN | 0.670 | 0.833 | <0.001 |
| Missouri | MO | 0.910 | 0.957 | <0.001 |
| Mississippi | MS | 0.936 | 0.968 | <0.001 |
| Montana | MT | 0.641 | -0.871 | <0.001 |
| North Carolina | NC | 0.961 | 0.982 | <0.001 |
| North Dakota | ND | 0.899 | 0.952 | <0.001 |
| Nebraska | NE | 0.727 | 0.864 | <0.001 |
| New Hampshire | NH | 0.696 | 0.847 | <0.001 |
| New Jersey | NJ | 0.881 | 0.943 | <0.001 |
| New Mexico | NM | 0.812 | 0.908 | <0.001 |
| Nevada | NV | 0.985 | 0.995 | <0.001 |
| New York | NY | 0.937 | 0.970 | <0.001 |
| Ohio | OH | 0.894 | 0.949 | <0.001 |
| Oklahoma | OK | 0.037 | 0.644 | 0.010 |
| Oregon | OR | 0.972 | 0.987 | <0.001 |
| Pennsylvania | PA | 0.670 | 0.833 | <0.001 |
| Rhode Island | RI | 0.049 | 0.342 | 0.212 |
| South Carolina | SC | 0.939 | 0.971 | <0.001 |
| South Dakota | SD | 0.071 | 0.371 | 0.173 |
| Tennessee | TN | 0.804 | 0.905 | <0.001 |
| Texas | TX | 0.916 | 0.960 | <0.001 |

| | | | | |
|---|---|---|---|---|
| Utah | UT | 0.817 | 0.904 | <0.001 |
| Virginia | VA | 0.967 | 0.984 | <0.001 |
| Vermont | VT | 0.578 | 0.780 | <0.001 |
| Washington | WA | 0.467 | 0.684 | 0.005 |
| Wisconsin | WI | 0.875 | 0.940 | <0.001 |
| West Virginia | WV | 0.537 | 0.755 | 0.001 |
| Wyoming | WY | 0.855 | 0.930 | <0.001 |
| Average | | 0.835105263 | 0.918473684 | |

*Table S11. The annual power generation of photovoltaic panels and wind turbines per kilowatt in China's provinces.[7,8] Related to STAR★Methods.*

| Province | kWh/kWp | kWh/MW | Province | kWh/kWp | kWh/MW |
|---|---|---|---|---|---|
| AH | 1163.2 | 2.19E+06 | JX | 1081.4 | 1.96E+06 |
| BJ | 1350.4 | 1.75E+06 | LN | 1434 | 2.99E+06 |
| CQ | 875 | 1.16E+06 | NM | 1738.1 | 3.29E+06 |
| FJ | 1115.1 | 1.95E+06 | NX | 1607.5 | 2.62E+06 |
| GD | 1147.6 | 1.71E+06 | QH | 1761.4 | 2.70E+06 |
| GS | 1667.5 | 3.53E+06 | SA | 1359 | 2.16E+06 |
| GX | 1002 | 1.77E+06 | SC | 1175.3 | 2.20E+06 |
| GZ | 918.9 | 1.85E+06 | SD | 1347.7 | 2.41E+06 |
| HA | 1192.3 | 1.26E+06 | SH | 1147.6 | 2.32E+06 |
| HB | 981.8 | 2.73E+06 | SX | 1412.1 | 2.57E+06 |
| HE | 1345.2 | 1.59E+06 | TJ | 1374.8 | 2.17E+06 |
| HL | 1501.4 | 2.93E+06 | XJ | 1584.1 | 3.08E+06 |
| HN | 1254.5 | 2.01E+06 | XZ | 1794.8 | 2.36E+06 |
| HU | 1101.7 | 1.03E+06 | YN | 1433.2 | 2.12E+06 |
| JL | 1475 | 2.56E+06 | ZJ | 1122.3 | 1.55E+06 |
| JS | 1215.5 | 2.53E+06 | | | |

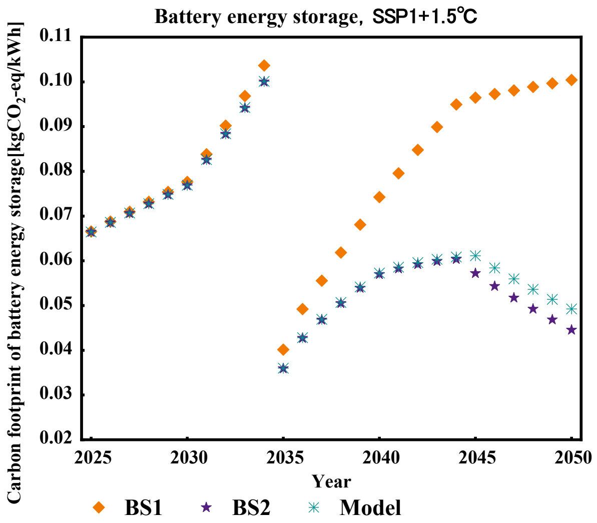


*Figure S1. Modeled carbon footprints under various methods for 1-kWh storage capacity of lithium battery in the electricity mix. Related to RESULTS.*

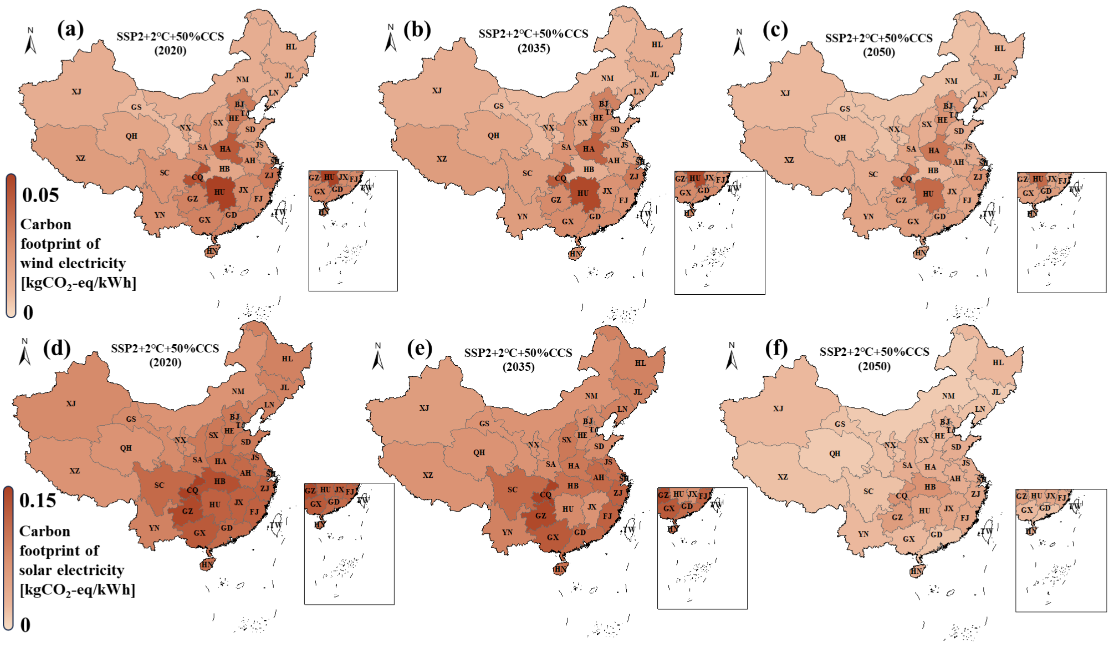


*Figure S2. The spatiotemporal comparison of the carbon footprint of wind and solar electricity in 2020, 2035, and 2050 under the SSP2+2°C+50%CCS scenario. Related to RESULTS.*

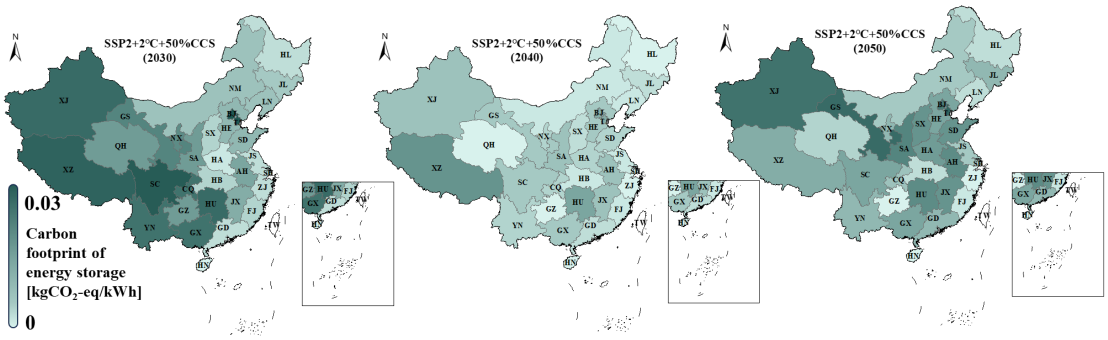


*Figure S3. The spatiotemporal comparison of the carbon footprint of energy storage in 2030, 2040, and 2050 under the SSP2+2°C+80%CCS scenario. Related to RESULTS.*

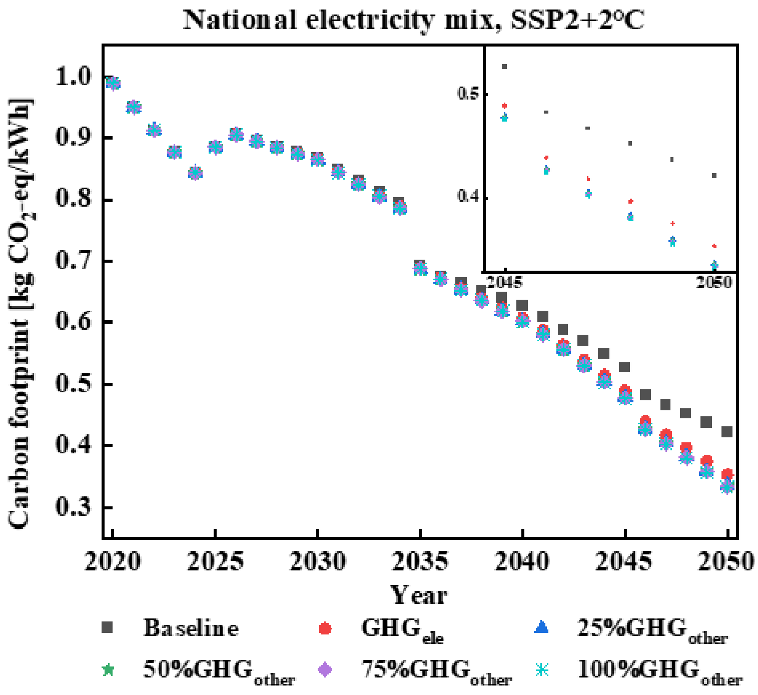


*Figure S4. Changes in the carbon footprint of national electricity mix: The impact of different proportions of $GHG_{other}$ (penetration rate in 2050) under the SSP2 scenario of 2°C. Related to DISCUSSION.*

References

1. Li, H., Cui, X., Hui, J., He, G., Weng, Y., Nie, Y., Wang, C., and Cai, W. (2021). Catchment-level water stress risk of coal power transition in China under 2℃ /1.5℃ targets. Applied Energy *294*. 10.1016/j.apenergy.2021.116986.
2. Mitigation Pathways Compatible with 1.5°C in the Context of Sustainable Development. (2022). In Global Warming of 1.5°C, pp. 93-174.

10.1017/9781009157940.004.

3. Wernet, G., Bauer, C., Steubing, B., Reinhard, J., Moreno-Ruiz, E., and Weidema, B. (2016). The ecoinvent database version 3 (part I): overview and methodology. The International Journal of Life Cycle Assessment *21*, 1218-1230. 10.1007/s11367-016-1087-8.
4. Volkart, K., Bauer, C., and Boulet, C. (2013). Life cycle assessment of carbon capture and storage in power generation and industry in Europe. International Journal of Greenhouse Gas Control *16*, 91-106. 10.1016/j.ijggc.2013.03.003.
5. Zhuo, Z., Du, E., Zhang, N., Nielsen, C.P., Lu, X., Xiao, J., Wu, J., and Kang, C. (2022). Cost increase in the electricity supply to achieve carbon neutrality in China. Nature Communications *13*. 10.1038/s41467-022-30747-0.
6. Cole, W., Carag, J.V., Brown, M., Brown, P., and Cohen, S. (2021). 2021 Standard Scenarios Report: A U.S. Electricity Sector Outlook. National Renewable Energy Lab. (NREL). NREL/TP6A4080641MainId:66372;UUID:7daed77cab2049afb0d416642fc4da87;MainAdminID:63368.
7. Solargis on behalf of the World Bank Group. (2022). Global Solar Atlas 2.7.
8. Thomas, J. (2021). Comparative analysis of wind atlases: Wind resource assessment of forested sites for wind power development. 10.13140/RG.2.2.23091.14880.
